# Supplementary material for: MetaQTL: a package of new computational methods for the meta-analysis of QTL mapping experiments
Source: BMC Bioinformatics. 2007 Feb 8;8:49. doi: 10.1186/1471-2105-8-49 (PMC1808479; doi:10.1186/1471-2105-8-49)
Supplement: Additional File 7 — MetaQTL Package : jar file and tutorial. This Zip archive contains both the MetaQTL JAR file and the files of the tutorial. [file 1471-2105-8-49-S7.zip › org.inra.metaqtl/doc/index-files/index-23.html]

X-Index


|  |  |  |
| --- | --- | --- |
| **Overview** \| Package \| Class \| Use \| **Tree** \| **Deprecated** \| **Index** \| **Help** | |  |
| **PREV LETTER**   **NEXT LETTER** | **FRAMES**    **NO FRAMES**     **All Classes** |  |


A B C D E F G H I K L M N O P Q R S T U V W X Y Z 

---


## **X**

**x** - Variable in class org.metaqtl.MetaQtlResult: The QTL positions. **x** - Variable in class org.metaqtl.TreeNode: **xc** - Variable in class org.metaqtl.TreeNode: **Xml2A** - Class in org.metaqtl.main: **Xml2A()** - Constructor for class org.metaqtl.main.Xml2A: **XmlBioEntitiesFactory** - Class in org.thalia.bio.entity.factory: Class Description Here **XmlBioEntitiesFactory()** - Constructor for class org.thalia.bio.entity.factory.XmlBioEntitiesFactory: **XmlBioEntityFactory** - Class in org.thalia.bio.entity.factory: Class Description Here **XmlBioEntityFactory()** - Constructor for class org.thalia.bio.entity.factory.XmlBioEntityFactory: **XmlGeneticMapFactory** - Class in org.thalia.bio.entity.factory: Class Description Here **XmlGeneticMapFactory()** - Constructor for class org.thalia.bio.entity.factory.XmlGeneticMapFactory: **XmlOntologyFactory** - Class in org.thalia.bio.entity.factory: **XmlOntologyFactory()** - Constructor for class org.thalia.bio.entity.factory.XmlOntologyFactory

---


|  |  |  |
| --- | --- | --- |
| **Overview** \| Package \| Class \| Use \| **Tree** \| **Deprecated** \| **Index** \| **Help** | |  |
| **PREV LETTER**   **NEXT LETTER** | **FRAMES**    **NO FRAMES**     **All Classes** |  |


A B C D E F G H I K L M N O P Q R S T U V W X Y Z 

---
